# Array expression meta-analysis of cancer stem cell genes identifies upregulation of *PODXL* especially in *DCC* low expression meningiomas

**DOI:** 10.1371/journal.pone.0215452

**Published:** 2019-05-13

**Authors:** Hans-Juergen Schulten, Deema Hussein

**Affiliations:** 1 Center of Excellence in Genomic Medicine Research, Department of Medical Laboratory Technology, Faculty of Applied Medical Sciences, King Abdulaziz University, Jeddah, Saudi Arabia; 2 King Fahad Medical Research Center, Faculty of Applied Medical Sciences, King Abdulaziz University, Jeddah, Saudi Arabia; 3 Faculty of Applied Medical Sciences, King Abdulaziz University, Jeddah, Saudi Arabia; University of South Alabama Mitchell Cancer Institute, UNITED STATES

## Abstract

**Background:**

Meningiomas are the most common intracranial tumors, with a subset of cases bearing a progressive phenotype. The DCC netrin 1 receptor (*DCC*) is a candidate gene for early meningioma progression. Cancer stem cell (CSC) genes are emerging as cancer therapeutic targets, as their expression is frequently associated with aggressive tumor phenotypes. The main objective of the study was to identify deregulated CSC genes in meningiomas.

**Materials and methods:**

Interrogating two expression data repositories, significantly differentially expressed genes (DEGs) were determined using *DCC* low *vs*. *DCC* high expression groups and WHO grade I (GI) *vs*. grade II + grade III (GII + GIII) comparison groups. Human stem cell (SC) genes were compiled from two published data sets and were extracted from the DEG lists. Biofunctional analysis was performed to assess associations between genes or molecules.

**Results:**

In the *DCC* low *vs*. *DCC* high expression groups, we assessed seven studies representing each between seven and 58 samples. The type I transmembrane protein podocalyxin like (*PODXL*) was markedly upregulated in *DCC* low expression meningiomas in six studies. Other CSC genes repeatedly deregulated included, *e*.*g*., BMP/retinoic acid inducible neural specific 1 (*BRINP1*), prominin 1 (*PROM1*), solute carrier family 24 member 3 (*SLC24A3*), rRho GTPase activating protein 28 (*ARHGAP28*), Kruppel like factor 5 (*KLF5*), and leucine rich repeat containing G protein-coupled receptor 4 (*LGR4*). In the GI *vs*. GII + GIII comparison groups, we assessed six studies representing each between nine and 68 samples. DNA topoisomerase 2-alpha (*TOP2A*) was markedly upregulated in GII + GIII meningiomas in four studies. Other CSC genes repeatedly deregulated included, *e*.*g*., *ARHGAP28* and *PODXL*. Network analysis revealed associations of molecules with, *e*.*g*., cellular development and movement; nervous system development and function; and cancer.

**Conclusions:**

This meta-analysis on meningiomas identified a comprehensive list of deregulated CSC genes across different array expression studies. Especially, PODXL is of interest for functional assessment in progressive meningiomas.

## Introduction

Based on ultrastructural and histologic similarities, meningiomas are described to originate from arachnoidal cells. Meningiomas account for approximately 30% of all primary intracranial brain tumors [1]. The majority of meningiomas are considered benign and are commonly treated by surgery; however, a minority of cases progress further or recur. The clinical behavior of meningiomas is assessed by using the grading system where benign meningiomas are classified as WHO grade I (GI) tumors and more aggressive meningiomas as atypical, grade II (GII) tumors, or as anaplastic, grade III (GIII) tumors. Molecular markers predicting aggressiveness of meningiomas are not well-known [2].

Cancer stem cells (CSCs) represent a population of cells that are implicated in cancer progression as well as chemo- and radioresistance [3–6]. Therefore, CSCs are emerging as therapeutic targets for improving treatment of aggressive types of cancer [7, 8]. The involvement of CSCs in different types of cancer is seemingly complex. Thus far, only a limited number of CSC genes and mechanisms have been identified and characterized in meningiomas [9–13]. Meningioma stromal mesenchymal stem-like cells, similar to bone marrow mesenchymal stem cells (SC), have been detected in meningiomas [3]. A recent immunohistochemical survey in GI meningiomas identified a number of SC markers, including OCT4, NANOG, SOX2, KLF4, and c-MYC, on microvessels that led to the suggestion that these vessels may be associated with meningioma initiation [14]. An immunohistochemical study detected higher expression of nestin (NES), SOX2, and prominin 1 (PROM1), also known as CD133, in more progressive meningiomas [9]. In meningioma cultures, cells with pleomorphic characteristics show markedly increased numbers of CSCs scoring positive for PROM1 and SOX2, or AGR2 and BMI1 [12]. Similarly, meningioma cells expressing PROM1 revealed a higher proliferation rate and the formation of tumorspheres [13].

In the present study, we focused on state-of-the art genome-wide array technology to identify CSC genes, which are deregulated in meningiomas. We performed a meta-analysis using publicly accessible array studies to investigate CSC expression profiles in meningiomas grouped according to their DCC netrin 1 receptor (*DCC*) expression levels or by tumor grade. DCC functions as a tumor suppressor in a various types including malignant astrocytomas where its reduced expression correlates with unfavorable prognosis [15]. In meningiomas, an array expression study has identified *DCC* as a candidate gene for early meningioma progression [16]. This finding was supported by the fact that 14 of 416 differentially expressed genes (DEGs) that were identified between *DCC* low and *DCC* high expression meningiomas were shared with 49 DEGs that were determined using a meta-analysis data set generated from a comparison of less *vs*. more progressive meningiomas [17]. In contrast, only four of 249 DEGs from the comparison GI *vs*. GII meningiomas were shared with the 49 DEGs of the meta-analysis data set. Array meta-analyses using post-statistical assessment of DEG data sets have been successfully conducted in cancer research [17, 18].

## Material and methods

### Interrogation of data base repositories

We interrogated the Gene Expression Omnibus (GEO) [19] and the ArrayExpress [20] repositories in March 2018 to retrieve published array expression data sets on meningiomas. The search query included meningioma AND expression AND array AND human.

### Transcriptome analysis

From the selected array studies, fulfilling the search criteria [21], binary CEL files containing the feature-level extraction output data were imported into the Transcriptome Analysis Console (TAC) version 4.0.1 (ThermoFisher Scientific Inc., Waltham, MA USA; previously branded Affymetrix microarray solutions) that includes the LIMMA (linear modeling for microarrays) package from Bioconductor [22]. In TAC, binary CEL files of each study were normalized utilizing robust multiarray analysis (RMA) algorithm. Array QC metrics analyses were performed using principal component analyses (PCA), 5`and 3`hybridization and labeling control graphs, and signal (log2) intensity bars. Lists of differentially expressed probe sets were generated based on the chosen parameters, including samples (N ≥ 2 per comparison arm) grouped according to the *DCC* expression values or tumor grade [16]. Samples that ranked in the lowest 30% or in the highest 50% of the *DCC* log2 intensity values were considered as *DCC* low or *DCC* high expression samples, respectively, with the exception of samples from submission GEO88720. This study employed a different array technology resulting in disproportionate log2 intensity values in comparison with other data sets and *DCC* high expression samples were considered to rank in the highest 30% of the intensity values. In all selected studies, the unitless *DCC* log2 intensity values ranged between 3.55 and 4.73. Samples with *DCC* medium expression levels (30% - 50%) were not considered for further DEG analysis. For U133 Plus 2.0 arrays, the 238914_at probe set was utilized to assess *DCC* expression levels of the samples. Files used for annotation were HuGene-1_0-st-v1.na36hg19.transcript.csv, HuGene-2_1-st-v1.na36hg19.transcript.csv, and HG-U133_Plus_2.na36.annot.csv. Threshold of significance for differentially expressed probe sets and for the subsequently generated DEGs was a *p*-value < 0.05 and fold change (FC) > 2. Where indicated, a false discovery rate (FDR)-adjusted *p*-value < 0.05 was employed. In general, the meta-analysis adhered to recommendations outlined in a practical guidance for meta-analysis of gene expression array data sets [21].

### CSC gene selection

We compiled a list of 366 human SC genes (S1 Table), including embryonic (E) SC and induced pluripotent (iP) SC genes, derived from two SC studies with intersecting gene lists [23, 24]. One study used in first instance amniocytes and the other study an N-glycoproteome as SC resource. The database for annotation, visualization and integrated discovery (DAVID) was employed to convert gene IDs to official gene symbols [25].

### Biofunctional analysis

Biological significance of identified CSC genes was interpreted using the Ingenuity Pathway Analysis software (IPA; build version 485516M; Ingenuity Systems, Redwood City, CA) that curates a comprehensive pathway knowledge base. Analysis settings comprised direct and indirect molecular associations. Significant associations between analyzed data set molecules and frameworks prebuilt or generated *de novo* by IPA were indicated by Fisher`s exact test *p*-values. The Molecule Activity Predictor was employed to predict expression effects of a molecule on further pathway/network molecules. Network analysis was performed to explore significance of fit, expressed as a score, between molecules of the uploaded data set and networks related to specific diseases and functions. The upstream analysis module was employed to evaluate in how far differences in target gene expression are effected by upstream regulators [26]. The gene ontology (GO) term finder LAGO (https://go.princeton.edu/LAGO/help.html) was employed to assess overrepresentation of GO terms [27].

## Results

**Fig 1:**
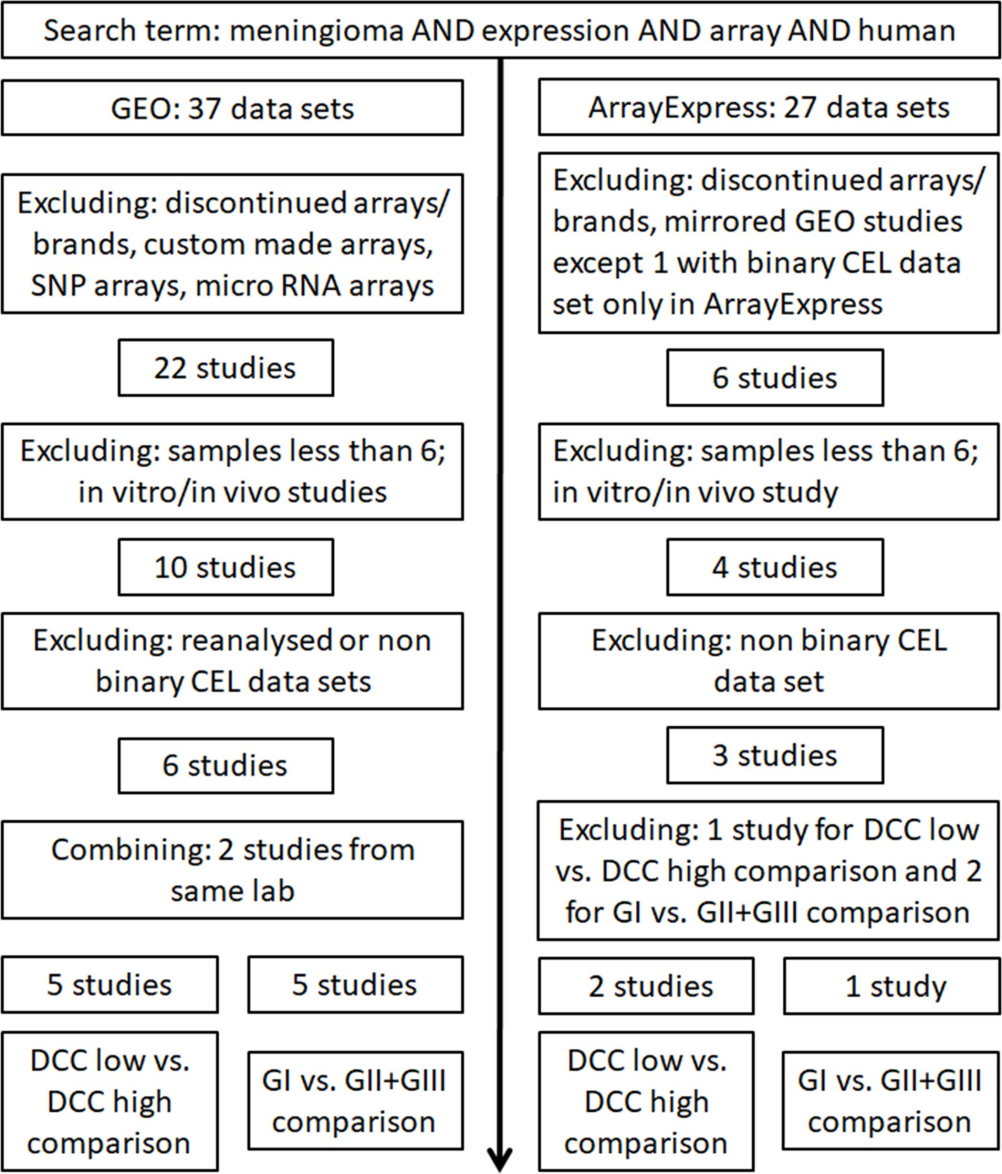
Flow chart for selecting CSC genes from meningioma array studies. Two publicly accessible array expression repositories were assessed to retrieve up-to-date array expression data sets on meningiomas.

**Fig 2:**
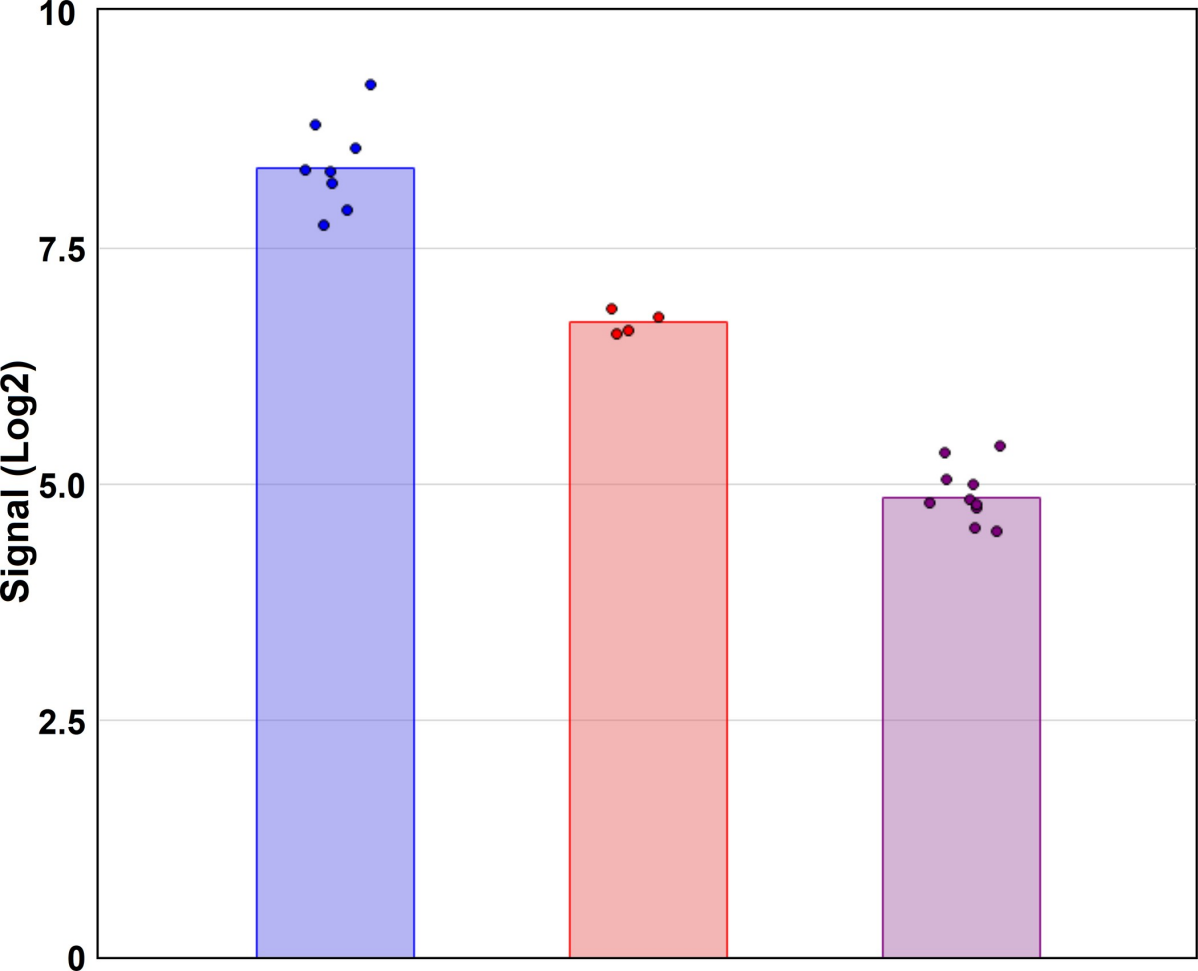
Categorization of GSE77259 and GSE100534 samples according to *DCC* expression values. DEGs in the DCC expression groups were compiled from *DCC* low *vs*. *DCC* high expression groups. Blue bars, *DCC* high expression samples; red bars, *DCC* medium expression samples; and purple bars, *DCC* low expression samples. Dots indicate expression values of individual samples. Significance between *DCC* low and *DCC* high expression groups is based on an FDR-adjusted *p*-value < 0.05.

Using the search query in GEO and ArrayExpress, identified 37 and 27 data sets, respectively (Fig 1). Exclusion criteria from the repositories included data sets that used custom-made, SNP or miRNA arrays; used discontinued array types or brands; contained < 6 meningioma samples or only *in vitro*/*in vivo* samples; comprised not the binary CEL files; or were reanalyzed data sets. Two data sets using HuGene 1.0 ST arrays were performed in the same laboratory and samples were pooled for subsequent analysis. In sum, eight studies from both repositories were selected for further analysis (Table 1). Two of eight studies used HuGene 1.0 ST arrays, one study used HuGene 2.1 ST arrays strips, and five studies employed U133 Plus 2.0 arrays. HuGene 1.0 ST arrays and HuGene 2.1 ST arrays strips represent whole transcript chips that interrogate gene expression levels on average with one probe per exon. In contrast, the U133 Plus 2.0 arrays interrogate expression levels primarily at the 3`-region of the genes. Seven selected studies met the criteria to establish sets of differentially expressed CSC genes from the *DCC* low *vs*. *DCC* high expression groups (Fig 2 and S1 Fig) whereas six studies met the criteria to establish sets of differentially expressed CSC genes from the GI *vs*. GII + GIII comparison groups [16, 28–35].

**Table 1:** Selected array expression studies for meta-analysis on CSC genes in meningiomas.

GEO/ArrayExpress accession number	Array type	Number of samples			
		*DCC* low	*DCC* high	GI	GII + GIII
**GSE54934**	HuGene 1.0 ST	10	8	20	2 + 0
**GSE77259, GSE100534**	HuGene 1.0 ST	10	8	16	5 + 1
**GSE88720**	HuGene 2.1 ST	9	2	12	2 + 0
**GSE16581**	U133 Plus 2.0	48	10	43	19 + 6
**GSE68015**	U133 Plus 2.0	4	3	5	4 + 0
**E-MTAB-1852**	U133 Plus 2.0	10	2	15	0
**E-GEOD-9438**	U133 Plus 2.0	13	5	N/A^1^	N/A
**E-MEXP-3586**	U133 Plus 2.0	17	1	15	4 + 0

N/A: not available.

### *DCC* low *vs*. *DCC* high expression groups

**Fig 3:**
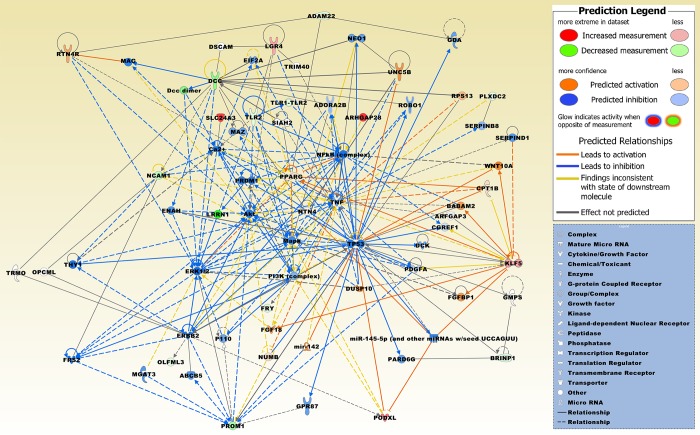
Top two merged networks of genes present in at least three different data sets in the *DCC* low *vs*. *DCC* high expression groups. These genes/molecules include, besides DCC, ADAM22, ARHGAP28, BRINP1, FRY, KLF5, LGR4, LRRN1, NCAM1, OLFML3, PLXDC2, PODXL, PROM1, SLC24A3, and TLR2. Integrative network molecules comprise, ATP binding cassette subfamily B member 5 (ABCB5), adenosine A2b receptor (ADORA2B), Akt, ADP ribosylation factor GTPase activating protein 3 (ARFGAP3), BRISC and BRCA1 A complex member 2 (BABAM2), Ca2+, cell growth regulator with EF-hand domain 1 (CGREF1), carnitine palmitoyltransferase 1B (CPT1B), Dcc dimer, deoxycytidine kinase (DCK), DS cell adhesion molecule (DSCAM), dual specificity phosphatase 10 (DUSP10), eukaryotic translation initiation factor 2A (EIF2A), ENAH, actin regulator (ENAH), erb-b2 receptor tyrosine kinase 2 (ERBB2), ERK1/2, fibroblast growth factor 18 (FGF18), fibroblast growth factor binding protein 1 (FGFBP1), fibroblast growth factor receptor substrate 2 (FRS2), guanine deaminase (GDA), guanine monophosphate synthase (GMPS), G protein-coupled receptor 87 (GPR87), myelin associated glycoprotein (MAG), Mapk, MYC associated zinc finger protein (MAZ), mannosyl (beta-1,4-)-glycoprotein beta-1,4-N-acetylglucosaminyltransferase (MGAT3), microRNA 142 (mir-142), miR-145-5p (and other miRNAs with seed UCCAGUU), neogenin 1 (NEO1), NFkB (complex), netrin 4 (NTN4), NUMB, endocytic adaptor protein (NUMB), opioid binding protein/cell adhesion molecule like (OPCML), P110, par-6 family cell polarity regulator gamma (PARD6G), platelet derived growth factor subunit A (PDGFA), PI3K (complex), peroxisome proliferator activated receptor gamma (PPARG), PR/SET domain 1 (PRDM1), roundabout guidance receptor 1 (ROBO1), ribosomal protein S13 (RPS13), reticulon 4 receptor (RTN4R), serpin family B member 8 (SERPINB8), serpin family D member 1 (SERPIND1), siah E3 ubiquitin protein ligase 2 (SIAH2), Thy-1 cell surface antigen (THY1), TLR1-TLR2, tumor necrosis factor (TNF), tumor protein p53 (TP53), tripartite motif containing 40 (TRIM40), tRNA methyltransferase O (TRMO), unc-5 netrin receptor B (UNC5B), and Wnt family member 10A (WNT10A). S3 Table contains the relationships of molecules.

**Fig 4:**
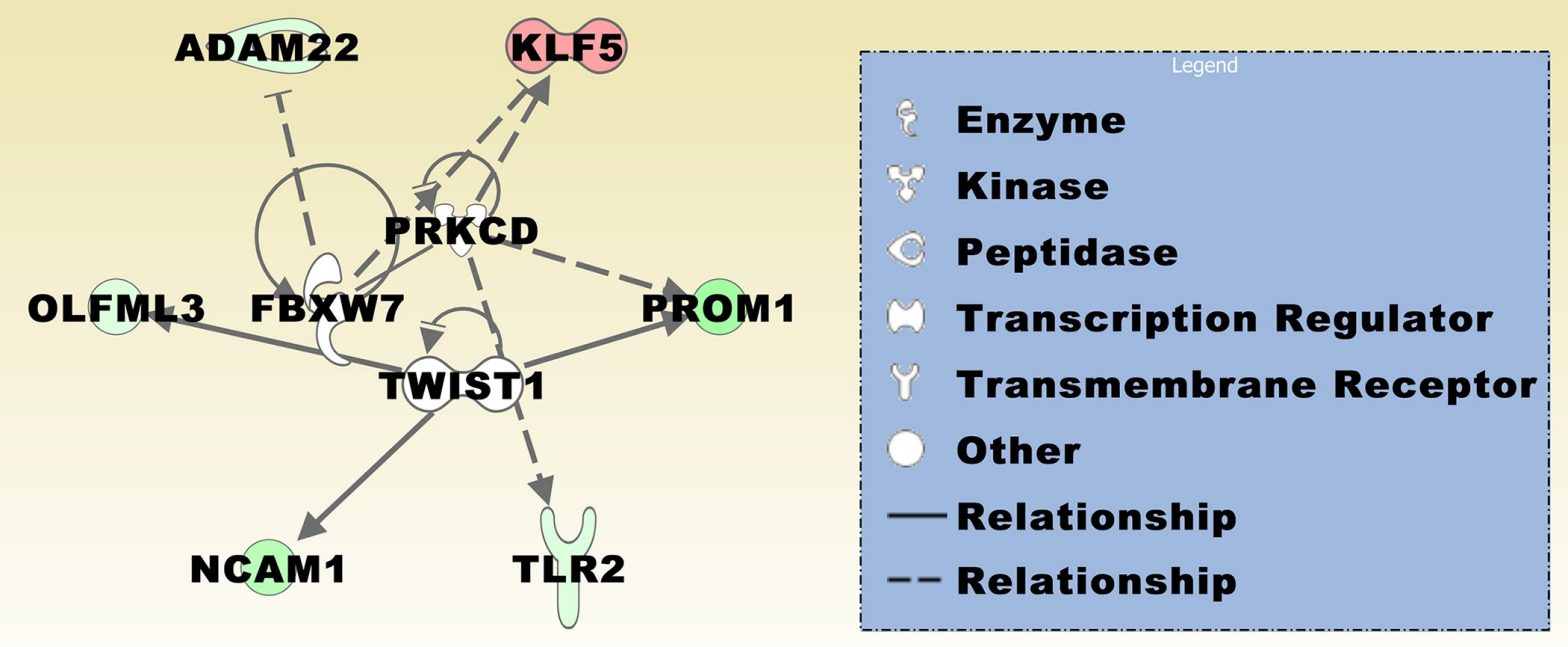
Upstream analysis of genes present in at least three different data sets from the *DCC* low *vs*. *DCC* high expression groups. The merged upstream regulator network contains the regulated genes/molecules, including PROM1, NCAM1, ADAM22, OLFML3, TLR2, and KLF5, and the three upstream regulators F-box and WD repeat domain containing 7 (FBXW7), protein kinase C delta (PRKCD), and twist family bHLH transcription factor 1 (TWIST1). See Fig 3 or Fig 5 for prediction legend.

In the *DCC* low *vs*. *DCC* high expression groups, podocalyxin like (*PODXL*) was significantly upregulated in *DCC* low expression meningiomas in six of seven studies (Table 2). The solute carrier family 24 member 3 (*SLC24A3*) was significantly upregulated in four studies. CSC genes that were upregulated in three studies include Rho GTPase activating protein 28 (*ARHGAP28*), Kruppel like factor 5 (*KLF5*), and leucine rich repeat containing G protein-coupled receptor 4 (*LGR4*). BMP/retinoic acid inducible neural specific 1 (*BRINP1*), and *PROM1* were significantly downregulated in *DCC* low expression meningiomas in five studies. Further CSC genes were downregulated in three or four studies, including ADAM metallopeptidase domain 22 (*ADAM22*), leucine rich repeats, calponin homology domain containing 4 (*LRRN1*), olfactomedin-like protein 3 (*OLFML3*), plexin domain containing 2 (*PLXDC2*), FRY microtubule binding protein (*FRY*), neural cell adhesion molecule 1 (*NCAM1*), and Toll-like receptor 2 (*TLR2*). Fig 3 displays a merged network of the category diseases and functions (Table 3) based on a number of genes including *DCC* and genes identified in at least three different data sets from the *DCC* low *vs*. *DCC* high expression groups (Table 2). Integrative network molecules presented in Fig 3 were derived from the knowledge base of the software application. Fig 4 illustrates a merged network of three upstream regulators that were significantly associated with a number of regulated genes (*p* ≤ 6.27E-04; Table 2).

**Table 2:** CSC genes significantly deregulated in *DCC* low *vs*. *DCC* high expression groups.

GEO/ArrayExpress accession number	CSC genes upregulated in *DCC* low *vs*. *DCC* high	CSC genes downregulated in *DCC* low *vs*. *DCC* high
**GSE54934**	LPAR3, PODXL^1^, SLC24A3^1^	ADAM22^1^, CNTFR, LRRN1^1^, OLFML3^1^, PODXL2^q^, PROM1^1^
**GSE77259, GSE100534**	LAMA1	BRINP1^1^, CNTFR, FRY^1^, GRID2, NCAM1^1^, OLFM2^q^, OLFML3^1^, PODXL2, PROM1^1^
**GSE88720**	GAL, KLF2, KLF4, KLF5^1^, LGR4^1^, PODXL^1^, RBX1, SLC24A3^1^	PLXDC2^1^, TLR2^1^
**GSE16581**	ADGRG2, ANOS1, ARHGAP28^1^^,^^q^, CCND1, CRABP1, EPHA7, GCNT2, KLF5^1^^,^^q^, LGR4^1^, LPAR3, PODXL^1^^,^^q^, SLC24A3^1^^,^^q^	ADAM22^1^^,^^q^, BRINP1^1^, CDK6, FRY^1^, LRRN1^1^^,^^q^, NCAM1^1^, OLFML3^1^, PLXDC2^1^^,^^q^, PROM1^1^^,^^q^, SFRP2^q^, SLC2A12, THY1
**GSE68015**	ARHGAP28^1^, JARID2, PODXL^1^	ADAM22^1^, BRINP1^1^, GABRB3, LRRN1^1^, NCAM1^1^, NTN1, THY1
**E-MTAB-1852**	PODXL^1^	ATP13A3, BRINP1^1^, IL27RA, KCNE3, MTF2, PLXDC2^1^, PROM1^1^, SLC2A12, SP1, TLR2^1^, ZIC3
**E-GEOD-9438**	APLP2^q^, ARHGAP28^1^^,^^q^, CCND1, CD44, FLT1, GCNT2, ID1, KITLG, KLF5^1^^,^^q^, LGR4^1^, PODXL^1^^,^^q^, SALL4, SLC24A3^1^^,^^q^	ADAM22^1^, AXIN2^q^, BRINP1^1^^,^^q^, CDH3, EED, FRY^1^, LRRN1^1^, NANOS1^q^, OLFML3^1^, PLXDC2^1^^,^^q^, PROM1^1^^,^^q^ SFRP2^q^, SLC15A2, TLR2^1^^,^^q^

^1^CSC genes significantly deregulated in at least three studies. GO annotations of these genes and of DCC are presented in S2 Table.

^q^CSC genes significantly differentially expressed based on an FDR-adjusted *p*-value (or *q*-value) < 0.05 and FC > 2.

**Table 3:** Top networks related to diseases and functions in the comparison groups.

Top networks	*DCC* low *vs*. *DCC* high	GII + GIII *vs*. GI
	score	score
Cellular movement, nervous system development and function, cancer	30	
Cellular development, nervous system development and function, cell death and survival	9	
Cellular assembly and organization, neurological disease, cancer		32
Cell death and survival, organismal functions, organismal injury and abnormalities		2

### GI *vs*. GII + GIII comparison groups

**Fig 5:**
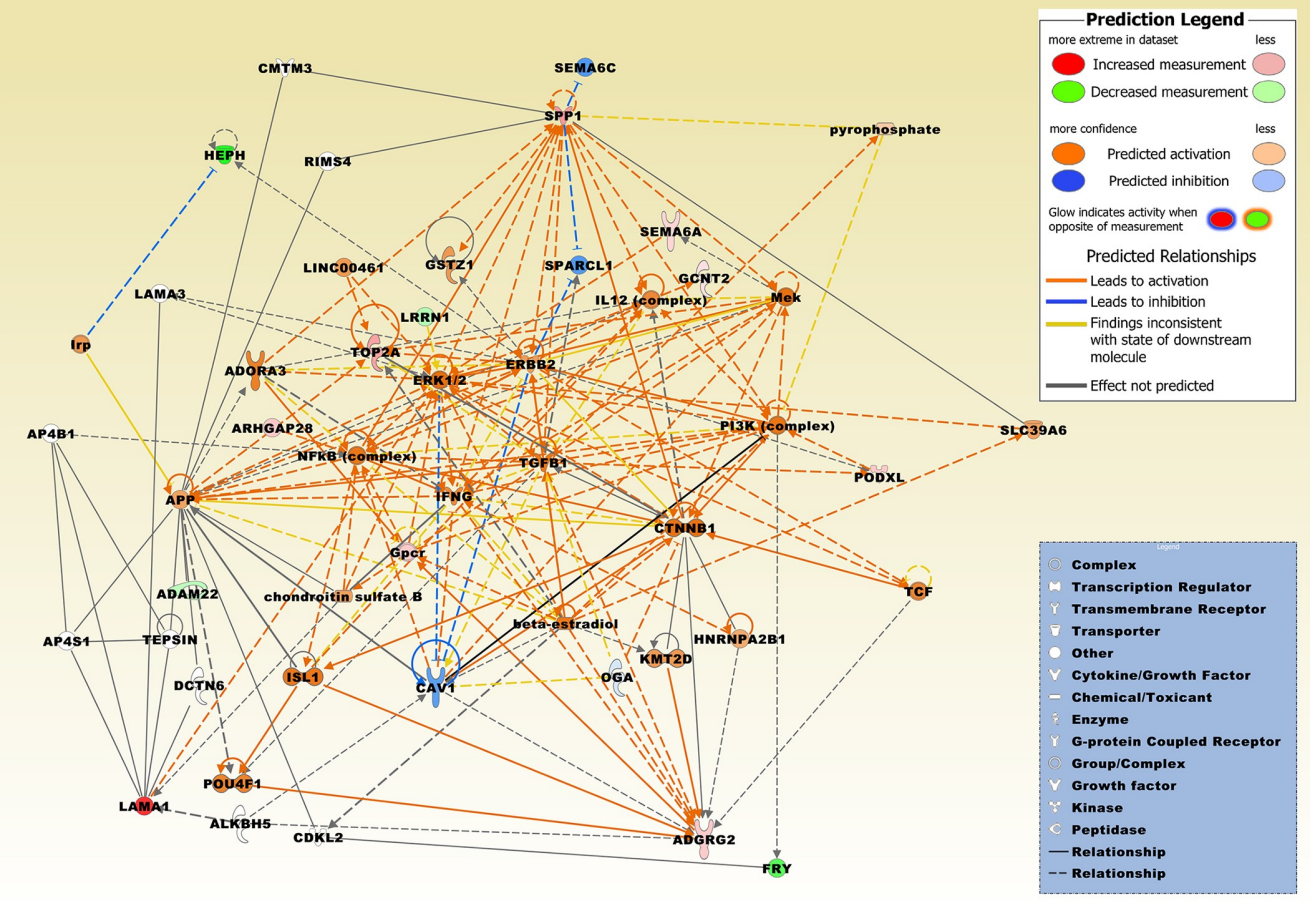
The top two merged networks of genes that were present in at least two different data sets from the GI *vs*. GII + GIII comparison groups. Genes/molecules include ADAM22, ADGRG2, ARHGAP28, FRY, GCNT2, HEPH, LAMA1, LRRN1, PODXL, SEMA6A, SPP1, and TOP2A. Integrative network molecules comprise, adenosine A3 receptor (ADORA3), alkB homolog 5, RNA demethylase (ALKBH5), adaptor related protein complex 4 subunit beta 1 (AP4B1), adaptor related protein complex 4 subunit sigma 1 (AP4S1), amyloid beta precursor protein (APP), beta-estradiol, caveolin 1 (CAV1), cyclin dependent kinase like 2 (CDKL2), chondroitin sulfate B, CKLF like MARVEL transmembrane domain containing 3 (CMTM3), catenin beta 1 (CTNNB1), dynactin subunit 6 (DCTN6), erb-b2 receptor tyrosine kinase 2 (ERBB2), ERK1/2, Gpcr, glutathione S-transferase zeta 1 (GSTZ1), heterogeneous nuclear ribonucleoprotein A2/B1 (HNRNPA2B1), interferon gamma (IFNG), IL12 (complex), Irp, ISL LIM homeobox 1 (ISL1), lysine methyltransferase 2D (KMT2D), laminin subunit alpha 3 (LAMA3), long intergenic non-protein coding RNA 461 (LINC00461), Mek, NFkB (complex), O-GlcNAcase (OGA), PI3K (complex), POU class 4 homeobox 1 (POU4F1), pyrophosphate, regulating synaptic membrane exocytosis 4 (RIMS4), semaphorin 6C (SEMA6C), solute carrier family 39 member 6 (SLC39A6), SPARC like 1 (SPARCL1), TCF, TEPSIN adaptor related protein complex 4 accessory protein (TEPSIN), and transforming growth factor beta 1 (TGFB1). S3 Table contains the relationships of molecules.

DNA topoisomerase 2-alpha (TOP2A) was significantly upregulated in GII + GIII meningiomas in four studies compared with GI (Table 4). Furthermore, adhesion G protein-coupled receptor G2 (*ADGRG2*), *ARHGAP28*, laminin subunit alpha 1 (*LAMA1*), glucosaminyl (N-acetyl) transferase 2 (I blood group) (*GCNT2*), *PODXL*, semaphorin 6A (*SEMA6A*), and secreted phosphoprotein 1 (*SPP1*) were upregulated and *ADAM22*, FRY microtubule binding protein (*FRY*), hephaestin (*HEPH*), and *LRRN1* were downregulated in two studies. Fig 5 displays a merged network of the category diseases and functions (Table 3) based on genes identified in at least two different data sets of the GI *vs*. GII + GIII comparison groups and on interconnecting molecules derived from the knowledge base of the software application.

**Table 4:** CSC genes significantly deregulated in GI *vs*. GII + GIII comparison groups.

GEO/ArrayExpress accession number	CSC genes upregulated in GII + GIII *vs*. GI	CSC genes downregulated in GII + GIII *vs*. GI
**GSE54934**	ARHGAP28^1^, PODXL^1^, SEMA6A^1^, TOP2A^1^	GJA1, LRRN1^1^
**GSE77259,** **GSE100534**	ANOS1, CD24, GPM6A, PODXL^1^, PTPRZ1, TOP2A^1^, ZIC3	CNTFR
**GSE88720**	CLUL1, GABRA3, GCNT2^1^	HEPH^1^
**GSE16581**	ADGRG2^1^, ARHGAP28^1^^,^^q^, CRABP1, EPHA7^q^, LAMA1^1^, OLFM4^q^, SPP1^1^^,^^q^, TOP2A^1^^,^^q^	ADAM22^1^^,^^q^, FRY^1^, HEPH^1^^,^^q^, LRRN1^1^, NPY1R, TLE1
**GSE68015**	CBX5, CCND2, CD44, DPP6, EZH2, KLF12, LAMA1^1^, LRIG1, MED13L, RBBP4, SALL1, SEMA6A^1^, SPP1^1^, TNFRSF21, TOP2A^1^	ADAM22^1^, CDH3, FRY^1^
**E-MEXP-3586**	ADGRG2^1^, GCNT2^1^, IL27RA, KITLG, NANOS1, SLC15A2	

^1^CSC genes significantly deregulated in at least two studies. GO annotations of these genes are presented in S2 Table.

^q^CSC genes significantly differentially expressed based on an FDR-adjusted *p*-value (or *q*-value) < 0.05 and FC > 2.

## Discussion

In our biostatistical meta-analysis, we identified CSC genes that, according to their expression profiles, are seemingly associated with *DCC* low expression meningiomas and, in their content, have not been reported before. We combined two resources to compile a list of SC genes that were used to identify differentially expressed CSC genes, which may exert a function in development and/or progression of meningiomas. One resource generated a reference list containing 250 human SC genes that were detected by transcriptome sequencing (RNA-seq) in cultured human amniocytes, or ESC and iPSC and were reported to have a functional relevance in SC maintenance. The authors commented that amniocytes inhere a unique SC identity and exist in a developmentally intermediate, hence uncommitted state [23]. The other resource employed cell surface capture technology and expression array assays to compile a list of 120 human PSC surface N-glycoproteins that were separated from those proteins that were also abundantly expressed in human fibroblasts or other non-diseased tissues [24]. The authors stated that the development of the cell surface capture technology enabled identification of proteins that otherwise are rarely detectable at the transcriptional level.

Evaluation of *DCC* low *vs*. *DCC* high expression groups generally identified more CSC genes than the GI *vs*. GII + GIII comparison groups. This can be partially attributed to the fact that a subset of benign meningiomas bear the capacity to evolve into more aggressive meningiomas and *DCC* expression levels are an appropriate molecular discriminator reflecting this characteristics [16]. In addition, it should be stated that the grading system for meningiomas remains suboptimal [36]. Array expression studies on meningiomas are preferentially conducted on a specific array platform [37], which is one of the reasons why this meta-analysis contained only studies from this brand; however, the selected array studies were performed with three different array types limiting the accumulation of array type specific DEGs. Furthermore, the selected studies were conducted in different geographical regions representing a heterogeneous population group.

### Upregulation of genes in *DCC* low expression and/or GII + GIII meningiomas

#### Upregulation of *PODXL* in *DCC* low expression and GII + GIII meningiomas

*PODXL* was upregulated in the current study, especially in *DCC* low expression groups. PODXL is a sialomucin and a type I transmembrane protein related to the hematopoietic SC factor CD34 and PODXL2. It exerts functions in regulating cell polarity through actin-dependent microvilli formation and was revealed to have an anti-adhesin capacity, which increases the adherence of cells to immobilized ligands and accelerates the rate of migration and cell-cell contacts [38, 39]. A meta-analysis on 12 studies revealed that high *PODXL* expression is significantly associated with worse overall survival in different cancer types [40]. Specifically, PODXL overexpression in MCF-7 breast cancer cells resulted in cell delamination from monolayers by perturbing cell junctions [41]. Furthermore, high PODXL expression was identified to be an independent factor for unfavorable prognosis in breast cancer patients. In mice, induced overexpression of PODXL led MCF-7 cell clusters to bud off from the primary tumor and invade mouse mammary gland stroma [42]. Moreover, in lung adenocarcinoma, PODXL overexpression induced epithelial-to-mesenchymal transition (EMT) [43].

Notably, expression of PODXL exerted a positive correlation with stem-like and EMT core signatures, and contributed to unfavorable prognosis in patients with colon cancer [44]. In addition, PODXL serves a critical role in cancer stemness, invasiveness and conferred chemotherapy resistance in HT29 and HCT15 colon cancer cells that expressed high PODXL levels. Compared with PODXL negative cells, PODXL positive cells express increased levels of progenitor/SC markers Musashi1, SOX2, and BMI1 [45]. The involvement of PODXL in glioblastoma multiforme (GBM) stem-like cell proliferation was demonstrated with PODXL positive cell populations in two GBM oncosphere lines that exhibited significantly elevated growth compared with PODXL negative cells [45]. In astrocytomas, high expression of PODXL is associated with unfavorable prognosis [46]. A newly generated monoclonal antibody directed against an extracellular epitope of PODXL was used in immumohistochemistry to specifically detect PODXL expressing normal renal cells, as well as colorectal, and breast cancer cells [47, 48]. Of notice, in a xenograft mouse model of colorectal adenocarcinoma, a human-mouse chimeric anti-PODXL antibody was shown to inhibit tumor growth of PODXL expressing cancer cells [49]. Preliminary findings of a current project in our labs, wherein we are studying the immunofluorescene staining patterns of PODXL and other stem cell markers in cell cultures derived from progressive meningiomas, let us suggest that PODXL is expressed in these entities. Therefore, based on its association with tumor progression in a number of cancer types, assessment of PODXL for its functional implications in more aggressive meningiomas is envisaged.

#### Upregulation of *TOP2A* in GII + GIII meningiomas

*TOP2A* was determined to be significantly upregulated in four GII + GIII groups but not in any *DCC* low expression group. Expression level of the cell cycle-dependent DNA topoisomerase peaks at the G2/M cell cycle phase. One of its major functions is decatenation of chromosomes during mitosis. Posttranslational modification of TOP2A, supporting its functions, include phosphorylation, ubiquitination, SUMOylation, and acetylation [50]. Interactions of TOP2A with cell cycle checkpoint protein MDC1 are associated with checkpoint activation and maintenance of genome stability. Another critical interactor of TOP2A is the tumor suppressor and DNA repair gene BRCA1. Abnormal activities of TOP2A and related molecules attribute to the instability of tumor genomes that is linked to tumor progression. *TOP2A* and *HER2* gene amplifications frequently coincide with a number of malignancies including breast, ovarian, pancreatic, and esophageal/gastroesophageal cancer [51]. An array meta-analysis revealed approximately 10-fold higher expression of *TOP2A* in GIII compared with GI meningiomas [52]. Due to its central role in decatenation, a number of anticancer compounds have been approved that are either TOP2A poisoning, such as doxorubicin, mitoxantrone, etoposide, and teniposide or TOP2A catalytic inhibitors, such as epirubicin, and idarubicin [50].

#### Upregulation of *SLC24A3*, *ARHGAP28*, *KLF5*, and *LGR4* in *DCC* low expression and/or GII + GIII meningiomas

SLC24A3, also known as NCKX3, is a member of the sodium/potassium/calcium exchangers. Its expression is most abundant in brain and smooth muscle [53, 54]. In tumorspheres of EBV positive nasopharyngeal carcinoma cells, *SLC24A3* was one of several CSC markers that were upregulated compared to corresponding monolayer cells [55]. Furthermore, tumorspheres were enriched in CD44 positive cells and these cells exhibited higher chemotherapeutic resistance. Knockdown of expression of transcription factor TFAP2C in hormone responsive breast carcinoma cells resulted in deregulation of a number of target genes including *SLC24A3*, which was downregulated [56]. *ARHGAP28* encodes a Rho GTPase activating protein (RhoGAP) that downregulates RhoA activity resulting in inhibition of actin stress fiber formation [57]. In mouse embryos, Arhgap28 exhibits a spatial and temporal expression pattern in tissues where a stiff extracellular matrix is assembled. *ARHGAP28* has been previously reported in array expression studies as a DEG and, using a platform not included in our meta-analysis, it was found to be upregulated in GII and, with statistical significance, in GIII compared with GI meningiomas [58]. In GBM-derived radioresistant tumor-initiating and PROM1-positive cell populations, *ARHGAP28* was upregulated upon treatment with the anticancer compound resveratrol that resulted in induction of apoptosis and elevated radiosensitivity through repression of STAT3 pathway signaling [59]. KLF5 is an evolutionary conserved zinc finger transcription factor that is known to participate in a number of key pathways including the Wnt, Ras, TGFβ, and Notch signaling pathways [60]. Knockdown experiments in murine embryonic SCs demonstrated that Klf5 exerts critical functions in inhibiting mesoderm differentiation [61]. Based on its implications in various signaling pathways and cancers, KLF5 has become a therapeutic target for cancer therapy development [60]. LGR4 is a G-protein-coupled transmembrane receptor for R-spondins and a regulator of the Wnt/β-catenin signaling pathway [62, 63]. Higher LGR4 expression correlates with unfavorable prognosis in breast and prostate cancer [62, 64]. In a xenograft mouse model, *LGR4* silencing in prostate cancer cells led to a delay of metastases and reduced expression of EMT markers [64].

### Downregulation of genes in *DCC* low expression and/or GII + GIII meningiomas

#### Downregulation of *BRINP1* and *PROM1* in *DCC* low expression meningiomas

BRINP1, alias DBC1, is a putative tumor suppressor gene that is a negative regulator of G1/S transition [65]. Reduced expression of *BRINP1* caused by different mechanisms, such as promoter hypermethylation, has been revealed in a number of tumor types including lymphoproliferative malignancies, non-small cell lung carcinomas, and astrocytomas [66–68]. Furthermore, in non-muscle-invasive bladder cancer, lower expression of BRINP1 is associated with unfavorable prognosis [69]. PROM1 is a pentaspan transmembrane glycoprotein that has been described by different research groups as a CSC factor in meningiomas [9, 10, 12, 13]. In our survey, *PROM1* was markedly downregulated in *DCC* low expression meningiomas in five array expression studies. Several controversies remain connected with the expression of the gene, its role as CSC marker, and its impact on tumor progression [70]. Multiple transcript variants of *PROM1* encoding different isoforms have been described [71]. Immunostaining in consecutive tissues using different PROM1 antibodies showed different protein expressions and characteristics [72]. GBM tumors, initiated directly from biopsies and engrafted intracerebrally into nude rats, expressed little or no PROM1 [73]. During serial passaging *in vivo*, the tumors gradually displayed increased PROM1 expression. In mice, GBM xenografts from PROM1-negative cells showed more proliferative and angiogenic features compared to that from PROM1-positive cells [74]. Previous work in meningiomas revealed different co-expression patterns of PROM1 and SOX2 in tissues compared to corresponding cell lines [12]. Whereas the average number of cells positive for both SOX2 and PROM1 significantly increased in GII + GIII meningioma cell lines, they significantly decreased in GII + GIII meningioma tissues compared to GI entities [10]. Taken together, these observations are compatible with a cyclic and microenvironmental-influenced expression of PROM1 [75, 76].

#### Downregulation of *ADAM22*, *LRRN1*, *OLFML3*, *PLXDC2*, *FRY*, *NCAM1*, and *TLR2* in *DCC* low expression and/or GII + GIII meningiomas

ADAM22 is a member of the ADAM family of disintegrins. Downregulation of *ADAM22* by siRNA in endocrine resistant cell populations resulted in impaired cell migration and reconstituted differentiation [77]. Similarly, treatment with recombinant LGI1, that serves as a ligand for ADAM22, impaired cell migration. In breast cancer, ADAM22 has been identified as an estrogen receptor independent predictor of disease-free survival and has been assessed as a target for endocrine resistant breast cancer therapy [78]. LRRN1 is associated with neuroepithelial boundary formations and its temporal expression changes during mammalian neural progenitor cell development [79, 80]. In embryonic SCs, *LRRN1*, alias NLRR1, was one of four genes markedly higher expressed than in fibroblasts [81] and in a xenograft mouse model, LRRN1 expression in neuroblastoma cells resulted in enhanced tumor growth [82]. OLFML3 has been identified as a proangiogenic factor [83]. It binds to BMP4 and in xenograft mice models of Lewis lung carcinoma cells, Olfml3 was expressed in tumor endothelial cells and pericytes. OLFML3 may constitute a target for antiangiogenic therapy as further *in vivo* experiments demonstrated that anti-Olfml3 antibodies impaired tumor growth and angiogenesis. Comparably higher expression of Olfml3 has been detected in the stroma transcriptome of osteoblastic bone metastases of prostate cancer [84]. PLXDC2 is a type I transmembrane protein that has been identified as component of network molecules implicated in modulating proliferation and differentiation of the developing nervous system. In embryonic neuroepithelial cells, PLXDC2 functions as a mitogen [85]. The evolutionarily conserved FRY protein is a microtubule binding factor that exerts critical functions in maintaining structural integrity of mitotic chromosomes in spindle bipolarity [86]. Especially, *FRY* silencing in *in vitro* experiments resulted in chromosome misalignment and multipolar spindle formation. NCAM1 is a cell adhesion molecule expressed preferentially in neurons, glia, skeletal muscles, and T cells. *In vitro* and *in vivo* experiments indicated that NCAM1 is necessary for EMT induction and maintenance and furthermore, high NCAM1 expression level was found to be associated with tumor invasion [87]. In a mouse model of intracerebral hemorrhage, increased expression of Tlr2 was observed, resulting in a proinflammatory gene profile with activation of Mmp9 [88]. Furthermore, blood-brain-barrier permeability was decreased in *Tlr2* knockout mice compared with wild type mice. *In vitro* microglial experiments demonstrated that Tlr2 induces random-like migration involving the Akt pathway [89].

In conclusion, there is compiling evidence that CSC are present in meningiomas and in this regard, a number of crucial SC markers are used to characterize CSC population; yet, the functional relevance of CSC in meningiomas requires further detailed studies [90]. Our meta-analysis identified a number of CSC genes that were repeatedly deregulated in the analyzed data sets in either or both of the comparison groups. Some of the identified CSC genes already represent valuable targets for specific inhibitors while others may represent new candidate genes. In particular, *POXDL*, which encodes a sialomucin and type I transmembrane protein and known to be involved in cell migration processes, is of interest for assessment of its functional implications in progressive meningiomas.

## Supporting information

S1 Fig Categorization of GEO data sets according to their *DCC* expression values. Array expression studies on meningiomas were used to extract expression of CSC genes from human SC and iPSC gene compilations. (A) GSE54934, (B) GSE88720, (C) GSE16581, (D) GSE68015, (E) E-MTAB-1852, and (F) E-GEOD-9438. Blue bars, *DCC* high expression samples; red bars, *DCC* medium expression samples; and purple bars, *DCC* low expression samples. Dots indicate expression values of individual samples. Except for GSE68015, significance between *DCC* low and *DCC* high expression groups is based on an FDR-adjusted *p*-value < 0.05. Of notice, using normalized, unlogged expression values and, with exception of GSE88720 samples, utilizing adapted, but unified, threshold values, the same gene lists for *DCC* low and *DCC* high expression samples were generated as specified in Table 2.
(TIF)

S1 Table Compiled list of SC genes. List is combined from two studies on SC genes [23, 24] and served to extract CSC genes from array expression studies on meningiomas.
(DOCX)

S2 Table Gene ontology annotations for the data set genes used to generate Figs 3 and 5. GO term finder LAGO was used to assess overrepresentation of GO terms.
(DOCX)

S3 Table Relationships of molecules of Figs 3 and 5. (DOCX)
