# Supplementary figures and images for: Array expression meta-analysis of cancer stem cell genes identifies upregulation of PODXL especially in DCC low expression meningiomas
Source: PLoS One. 2019 May 13;14(5):e0215452. doi: 10.1371/journal.pone.0215452 (PMC6513070; doi:10.1371/journal.pone.0215452)

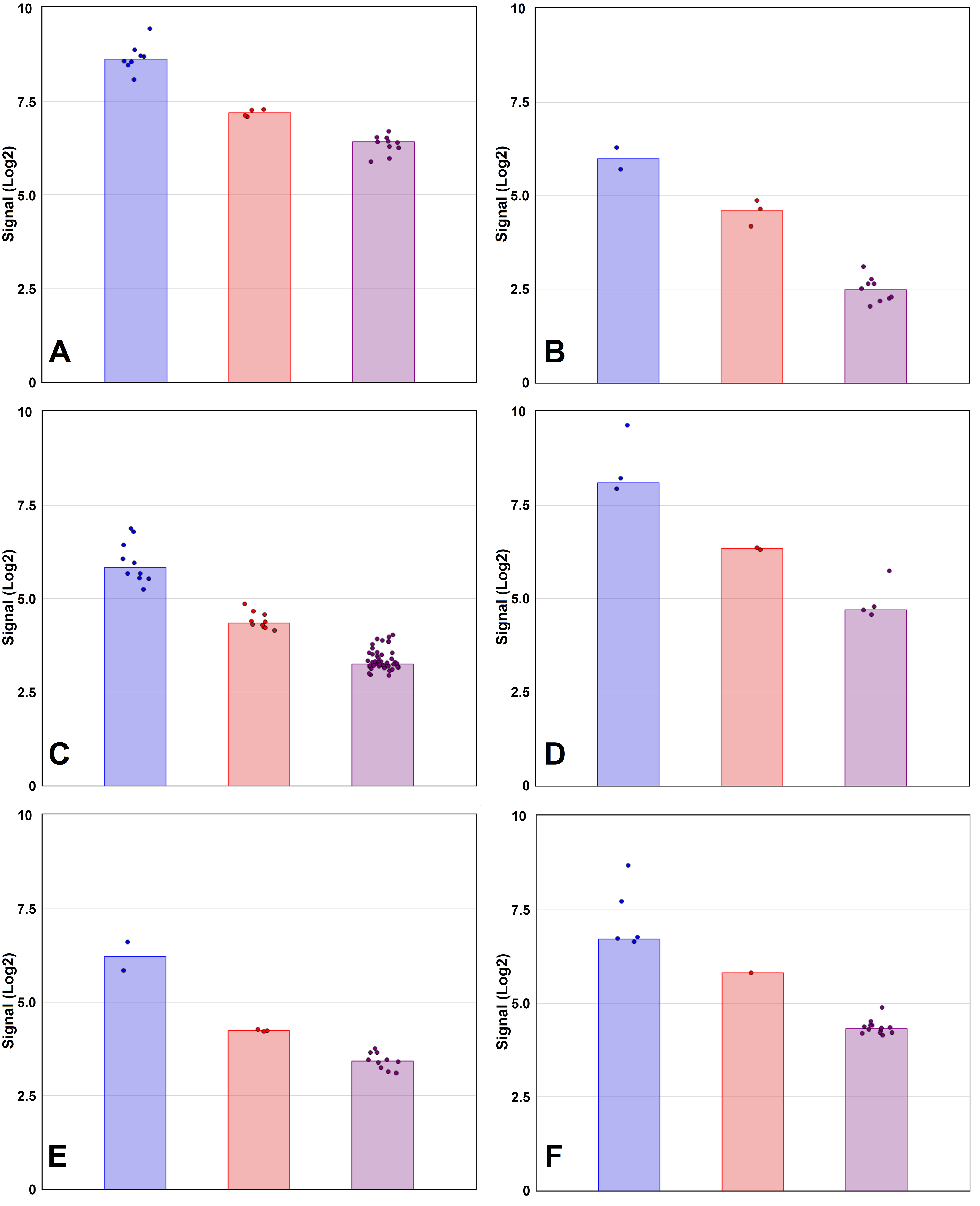

Supplement: S1 Fig — Array expression studies on meningiomas were used to extract expression of CSC genes from human SC and iPSC gene compilations. (A) GSE54934, (B) GSE88720, (C) GSE16581, (D) GSE68015, (E) E-MTAB-1852, and (F) E-GEOD-9438. Blue bars, DCC high expression samples; red bars, DCC medium expression samples; and purple bars, DCC low expression samples. Dots indicate expression values of individual samples. Except for GSE68015, significance between DCC low and DCC high expression groups is based on an FDR-adjusted p-value < 0.05. Of notice, using normalized, unlogged expression values and, with exception of GSE88720 samples, utilizing adapted, but unified, threshold values, the same gene lists for DCC low and DCC high expression samples were generated as specified in Table 2. (TIF) [file pone.0215452.s001.tif]
