# Supplementary material for: Array expression meta-analysis of cancer stem cell genes identifies upregulation of PODXL especially in DCC low expression meningiomas
Source: PLoS One. 2019 May 13;14(5):e0215452. doi: 10.1371/journal.pone.0215452 (PMC6513070; doi:10.1371/journal.pone.0215452)
Supplement: S1 Table — List is combined from two studies on SC genes [23, 24] and served to extract CSC genes from array expression studies on meningiomas. (DOCX) [file pone.0215452.s002.docx]

**S1 Table. Compiled list of SC genes.** The list is combined from two studies on SC genes [23, 24] and served to extract CSC genes from array expression studies on meningiomas.

| ACVR2B, ADAM22, ADCYAP1R1, ADGRG2, ADGRV1, ALCAM, AMIGO3, ANOS1, APLP2, ARHGAP28, ARID1B, ASH2L, ASTN1, ATP13A3, ATP6V0A2, AXIN2, B4GALT3, BMI1, BRINP1, BRX1, C11orf87, CACNG8, CALCR, CBX1, CBX5, CCKBR, CCNA1, CCND1, CCND2, CCNE1, CCNF, CD24, CD44, CD83, CD9, CDH3, CDK2, CDK4, CDK6, CDKN1B, CDYL, CELSR2, CHD1, CHD7, CHST6, CKS1B, CLDN6, CLECL1, CLUL1, CNOT1, CNOT2, CNOT3, CNTFR, CNTN2, COPS2, COPS4, CPSF3, CPSF3L, CRABP1, DAZAP1, DNMT3B, DPEP1, DPP6, DPPA2, DPPA3, DPPA4, DPPA5, DPY30, E2F1, EED, EFNA3, EHMT2, EIF2B1, EIF2B2, EIF2B3, EIF2S2, ELFN1, ENTPD2, EPCAM, EPHA7, ERAS, ESRRB, EWSR1, EZH1, EZH2, FAM216A, FBXO15, FGF13, FGF4, FGFR4, FKRP, FLT1, FLT3, FOXD3, FOXH1, FRY, FTH1, FUT4, FUT9, GABRA3, GABRA5, GABRB3, GAL, GBX2, GCNT2, GDF3, GDPD2, GJA1, GLI1, GLI2, GLI3, GLIS1, GNL3, GNPTAB, GPM6A, GPR20, GRB7, GRID2, H2AFZ, HAS2, HCFC1, HEPH, HERC5, HESX1, HIRA, HMGA1, HPN, HSPA4, HSPB1, HTR2C, ID1, ID2, IGF2BP1, IGSF1, IGSF21, IGSF9, IL17RD, IL27RA, IL4R, ING5, INHBE, ITGA6, JARID2, KAT2A, KAT5, KAT6A, KCNE3, KCNE5, KDM1A, KDM3A, KDM4A, KDM4C, KDM5B, KDR, KIT, KITLG, KLF12, KLF2, KLF4, KLF5, KMT2A, KMT2C, KMT2D, KMT2E, L1TD1, LAMA1, LAMB2, LEFTY1, LEFTY2, LGR4, LIN28A, LIN28B, LINGO1, LPAR3, LPAR4, LRIG1, LRP4, LRRN1, LY6E, MAPK1, MAX, MC5R, MCM2, MCRS1, MED1, MED10, MED12, MED13, MED13L, MED14, MED17, MED19, MED24, MED28, MEGF10, METAP2, MFSD11, MGA, MICB, MMGT1, MMP15, MSI1, MT1A, MT2A, MTF2, MTHFD1, MYBL2, MYC, MYCN, NACC1, NANOG, NANOS1, NCAM1, NCLN, NCOA2, NCOA3, NFRKB, NLGN4X, NODAL, NPR1, NPY1R, NR0B1, NR6A1, NRXN1, NTN1, NTS, OLFM2, OLFM4, OLFML3, ONECUT2, OTX1, OTX2, PAF1, PCDH11X, PCGF6, PCID2, PCNA, PHC1, PHC2, PHC3, PIM2, PLA2G3, PLXDC2, PODXL, PODXL2, POU5F1, PPP1R3D, PRDM14, PRDM16, PRDM5, PRMT6, PROM1, PTPRD, PTPRU, PTPRZ1, PUM1, PUM2, RAD21, RB1, RBBP4, RBBP5, RBBP7, RBBP9, RBL2, RBX1, REST, RIF1, RING1, RNF2, RTF1, SALL1, SALL4, SEMA4A, SEMA4B, SEMA6A, SETDB1, SETDB2, SF3A1, SF3A3, SFRP2, SIRT2, SKIL, SLC15A1, SLC15A2, SLC24A3, SLC2A12, SLC30A4, SLC38A2, SLC38A5, SLC38A9, SLC43A1, SLC6A5, SLC7A3, SLITRK4, SMAD1, SMAD2, SMAD3, SMARCA4, SMARCA5, SMARCAD1, SMARCB1, SMARCC1, SMARCD1, SMC1A, SMO, SOX2, SOX3, SP1, SPINT1, SPP1, SRRT, ST14, STAG1, STAT3, STX1B, SUB1, SUV39H2, SUZ12, TAF2, TAF7, TCF3, TCF7L1, TCL1A, TDGF1, TERF1, TERT, TGIF1, THAP11, THY1, TLE1, TLR2, TMEM114, TMEM132B, TMEM200B, TMEM63A, TMEM63C, TNFRSF10C, TNFRSF21, TNFRSF8, TNFSF11, TOP2A, TRIM16, TRIM24, TRIM28, UNC5D, UTF1, VSIG10, VSIG10L, WBSCR17, WDR18, WDR5, WNT2B, WNT8A, XPO7, YY1, ZFHX3, ZFP41, ZFP42, ZFX, ZIC2, ZIC3, ZIC5, ZNF143, ZNF219, ZNF281, ZSCAN10 |
| --- |
