# Supplementary material for: Array expression meta-analysis of cancer stem cell genes identifies upregulation of PODXL especially in DCC low expression meningiomas
Source: PLoS One. 2019 May 13;14(5):e0215452. doi: 10.1371/journal.pone.0215452 (PMC6513070; doi:10.1371/journal.pone.0215452)
Supplement: S2 Table — GO term finder LAGO was used to assess overrepresentation of GO terms. (DOCX) [file pone.0215452.s003.docx]

**S2 Table. Gene Ontology annotations for data set genes used to generate Fig 3 and Fig 5.** GO term finder LAGO was used to assess overrepresentation of GO terms.

| GO ID | | Term | Bonferroni-  corrected  *p* -value | Annotated genes |
| --- | --- | --- | --- | --- |
| Fig 3 data set genes | |  |  |  |
|  | GO:0032534 | regulation of microvillus assembly | 3.50E-03 | KLF5, PODXL |
|  | GO:0061005 | cell differentiation involved in kidney development | 3.73E-03 | LGR4, PODXL, PROM1 |
|  | GO:0032835 | glomerulus development | 5.13E-03 | LGR4, PODXL, PROM1 |
|  | GO:0007275 | multicellular organism development | 9.99E-03 | ADAM22, BRINP1, DCC, FRY, KLF5, LGR4, LRRN1, NCAM1, OLFML3, PODXL, PROM1, TLR2 |
| Fig 5 data set genes | |  |  |  |
|  | GO:0007162 | negative regulation of cell adhesion | 4.64E-03 | ADAM22, GCNT2, PODXL, SEMA6A |
|  | GO:0030155 | regulation of cell adhesion | 8.95E-03 | ADAM22, GCNT2, LAMA1, PODXL, SEMA6A |
