# Supplementary material for: Array expression meta-analysis of cancer stem cell genes identifies upregulation of PODXL especially in DCC low expression meningiomas
Source: PLoS One. 2019 May 13;14(5):e0215452. doi: 10.1371/journal.pone.0215452 (PMC6513070; doi:10.1371/journal.pone.0215452)
Supplement: S3 Table — (DOCX) [file pone.0215452.s004.docx]

**S3 Table. Relationships of molecules of Figs 3 and 5.**

| **Figure** | **Relationship from molecule(s)** | **Relationship**  **to**  **molecule(s)** | **Relationship**  **type** |
| --- | --- | --- | --- |
| Figure 3 |  |  |  |
|  | ADORA2B | Akt, NFkB (complex) | activation |
|  | ADORA2B | TNF | expression |
|  | ADORA2B | TNF | localization |
|  | ADORA2B | DCC | protein-protein interactions |
|  | ADORA2B | DCC | regulation of binding |
|  | ARHGAP28 | NFkB (complex) | activation |
|  | Akt | Akt, ERK1/2, NFkB (complex) | activation |
|  | Akt | Akt, Ca2+, NFkB (complex), PPARG, TLR2, TNF, TP53 | expression |
|  | Akt | Akt | inhibition |
|  | Akt | Ca2+, TNF, TP53 | localization |
|  | Akt | TP53 | molecular cleavage |
|  | Akt | Akt, ERK1/2 | phosphorylation |
|  | Akt | Akt, ERK1/2 | protein-protein interactions |
|  | Akt | NFkB (complex), TNF | regulation of binding |
|  | Akt | Akt | translocation |
|  | Akt | TP53 | ubiquitination |
|  | BABAM2 | NFkB (complex) | activation |
|  | BRINP1 | BRINP1 | regulation of binding |
|  | Ca2+ | Akt, EIF2A, ERK1/2, Mapk, NFkB (complex), PI3K (complex) | activation |
|  | Ca2+ | Ca2+ | chemical-chemical interactions |
|  | Ca2+ | PI3K (complex), TP53 | chemical-protein interactions |
|  | Ca2+ | Ca2+, MAG, PPARG, TNF | expression |
|  | Ca2+ | Ca2+, TNF | localization |
|  | Ca2+ | TP53 | molecular cleavage |
|  | Ca2+ | Akt | phosphorylation |
|  | Ca2+ | EIF2A, ERK1/2, Mapk | phosphorylation |
|  | Ca2+ | Ca2+, NFkB (complex), TP53 | regulation of binding |
|  | Ca2+ | TNF | transcription |
|  | Ca2+ | Ca2+ | translocation |
|  | DCC | ENAH, ERK1/2 | activation |
|  | DCC | MAG | expression |
|  | DCC | DCC | molecular cleavage |
|  | DCC | ADORA2B, DCC | protein-protein interactions |
|  | DCC | NCAM1 | regulation of binding |
|  | DCC | DCC | ubiquitination |
|  | DCK | DCK | protein-protein interactions |
|  | DSCAM | DCC | protein-protein interactions |
|  | DUSP10 | ERK1/2, TP53 | activation |
|  | DUSP10 | KLF5, TNF | expression |
|  | DUSP10 | Mapk | inhibition |
|  | DUSP10 | TNF | localization |
|  | DUSP10 | Mapk, TP53 | phosphorylation |
|  | DUSP10 | TP53 | transcription |
|  | Dcc dimer | Ca2+, ERK1/2 | activation |
|  | Dcc dimer | DCC | membership |
|  | EIF2A | EIF2A, PPARG | activation |
|  | EIF2A | EIF2A | phosphorylation |
|  | EIF2A | DCC | protein-protein interactions |
|  | ENAH | Akt, ERK1/2, Mapk | activation |
|  | ERBB2 | Akt, ERBB2, ERK1/2, FRS2, Mapk, NFkB (complex), PI3K (complex), PPARG | activation |
|  | ERBB2 | Akt, ERBB2, Mapk, NFkB (complex), PPARG, PROM1, THY1, TNF, TP53 | expression |
|  | ERBB2 | Akt | inhibition |
|  | ERBB2 | OLFML3, PODXL | localization |
|  | ERBB2 | ERBB2, TP53 | molecular cleavage |
|  | ERBB2 | Akt, ERBB2, ERK1/2, FRS2, Mapk | phosphorylation |
|  | ERBB2 | ABCB5, ERBB2, Mapk, PI3K (complex), TP53 | protein-protein interactions |
|  | ERBB2 | ERBB2, NFkB (complex) | regulation of binding |
|  | ERBB2 | TP53 | transcription |
|  | ERBB2 | ERBB2 | ubiquitination |
|  | ERK1/2 | Akt, ERK1/2, Mapk, NFkB (complex), PPARG, TNF, TP53 | activation |
|  | ERK1/2 | TNF | expression |
|  | ERK1/2 | FRS2, PPARG | inhibition |
|  | ERK1/2 | TNF | localization |
|  | ERK1/2 | Akt, ERK1/2, FRS2, NFkB (complex), PPARG, TP53 | phosphorylation |
|  | ERK1/2 | Akt, ERK1/2, TP53 | protein-protein interactions |
|  | ERK1/2 | ERK1/2, TNF | regulation of binding |
|  | ERK1/2 | ERK1/2 | translocation |
|  | FGF18 | ERK1/2, Mapk | activation |
|  | FGF18 | ERK1/2 | phosphorylation |
|  | FGFBP1 | Mapk | activation |
|  | FGFBP1 | Mapk | phosphorylation |
|  | FGFBP1 | FGFBP1 | protein-protein interactions |
|  | FRS2 | Akt, ERK1/2, FRS2, Mapk, PI3K (complex) | activation |
|  | FRS2 | ERBB2, PROM1 | expression |
|  | FRS2 | ERK1/2, FRS2 | phosphorylation |
|  | FRS2 | FRS2 | regulation of binding |
|  | GDA | ADAM22 | protein-protein interactions |
|  | GDA | GDA | regulation of binding |
|  | GMPS | GMPS | localization |
|  | GMPS | BRINP1 | protein-protein interactions |
|  | GPR87 | PROM1 | expression |
|  | KLF5 | PPARG | activation |
|  | KLF5 | BABAM2, CGREF1, CPT1B, FGF18, FGFBP1, KLF5, PDGFA, WNT10A | expression |
|  | KLF5 | FGFBP1 | localization |
|  | KLF5 | KLF5 | molecular cleavage |
|  | KLF5 | FGFBP1, PDGFA, PPARG | protein-DNA interactions |
|  | KLF5 | ARFGAP3, DCK, PPARG, TP53 | protein-protein interactions |
|  | KLF5 | KLF5, TP53 | regulation of binding |
|  | KLF5 | FGFBP1, PDGFA | transcription |
|  | KLF5 | KLF5 | ubiquitination |
|  | LGR4 | Akt | activation |
|  | LGR4 | TNF | expression |
|  | LGR4 | Akt | phosphorylation |
|  | LGR4 | DSCAM, LGR4 | protein-protein interactions |
|  | LRRN1 | ERK1/2 | activation |
|  | MAG | Ca2+, RTN4R | expression |
|  | MAG | Akt | inhibition |
|  | MAG | RTN4R | protein-protein interactions |
|  | MAZ | MAZ, PPARG | expression |
|  | MAZ | MAZ | protein-DNA interactions |
|  | MAZ | DCC | protein-protein interactions |
|  | MGAT3 | ERK1/2 | activation |
|  | MGAT3 | PROM1 | expression |
|  | Mapk | Akt, ERK1/2, FRS2, MAZ, NFkB (complex), TP53 | activation |
|  | Mapk | TNF | expression |
|  | Mapk | PPARG | inhibition |
|  | Mapk | Ca2+ | localization |
|  | Mapk | Akt, FRS2, MAZ, PPARG, TP53 | phosphorylation |
|  | Mapk | ADORA2B, ERBB2 | protein-protein interactions |
|  | Mapk | PI3K (complex) | regulation of binding |
|  | Mapk | TNF | transcription |
|  | Mapk | Mapk | translocation |
|  | NCAM1 | Mapk, NFkB (complex) | activation |
|  | NCAM1 | Ca2+, NCAM1, THY1 | expression |
|  | NCAM1 | NCAM1 | molecular cleavage |
|  | NCAM1 | FRS2, NCAM1 | protein-protein interactions |
|  | NCAM1 | NCAM1 | regulation of binding |
|  | NEO1 | NTN4 | activation |
|  | NEO1 | TNF | localization |
|  | NEO1 | DCC, NTN4, TNF | protein-protein interactions |
|  | NFkB (complex) | NFkB (complex), PI3K (complex), TP53 | activation |
|  | NFkB (complex) | ADORA2B, CGREF1, ERK1/2, NCAM1, PPARG, PRDM1, TLR2, TNF, TP53, WNT10A | expression |
|  | NFkB (complex) | PPARG | inhibition |
|  | NFkB (complex) | Ca2+, TNF | localization |
|  | NFkB (complex) | ERBB2 | molecular cleavage |
|  | NFkB (complex) | ERBB2, TLR2, TNF, TP53 | protein-DNA interactions |
|  | NFkB (complex) | PPARG | protein-protein interactions |
|  | NFkB (complex) | NFkB (complex), TP53 | regulation of binding |
|  | NFkB (complex) | TNF, TP53 | transcription |
|  | NFkB (complex) | NFkB (complex) | translocation |
|  | NTN4 | Akt, ERK1/2 | activation |
|  | NTN4 | Akt, ERK1/2 | phosphorylation |
|  | NTN4 | DCC, NEO1, NTN4 | protein-protein interactions |
|  | NUMB | Akt | activation |
|  | NUMB | PROM1, TP53 | expression |
|  | NUMB | NUMB | molecular cleavage |
|  | NUMB | TP53 | protein-protein interactions |
|  | NUMB | NUMB, TP53 | regulation of binding |
|  | NUMB | NUMB, TP53 | ubiquitination |
|  | OPCML | ERBB2, NCAM1 | protein-protein interactions |
|  | P110 | Akt, ERK1/2 | activation |
|  | P110 | P110 | localization |
|  | P110 | Akt | phosphorylation |
|  | PARD6G | BRINP1 | protein-protein interactions |
|  | PDGFA | Akt, Mapk | activation |
|  | PDGFA | PROM1 | expression |
|  | PDGFA | Akt | phosphorylation |
|  | PDGFA | PDGFA | protein-protein interactions |
|  | PI3K (complex) | Akt, ERK1/2, Mapk, NFkB (complex), PI3K (complex), TLR2 | activation |
|  | PI3K (complex) | Ca2+ | chemical-protein interactions |
|  | PI3K (complex) | Akt, Ca2+, FRY, Mapk, NFkB (complex), PRDM1, TNF, TP53 | expression |
|  | PI3K (complex) | Akt | inhibition |
|  | PI3K (complex) | Akt, Ca2+, TNF | localization |
|  | PI3K (complex) | FRS2, P110 | membership |
|  | PI3K (complex) | Akt, ERK1/2, Mapk, NFkB (complex), PI3K (complex) | phosphorylation |
|  | PI3K (complex) | Akt, ERBB2 | protein-protein interactions |
|  | PI3K (complex) | NFkB (complex), PI3K (complex) | regulation of binding |
|  | PI3K (complex) | NFkB (complex) | transcription |
|  | PODXL | Mapk, PI3K (complex) | activation |
|  | PPARG | Akt, ERK1/2, NFkB (complex), PI3K (complex), PPARG, TP53 | activation |
|  | PPARG | Akt, CPT1B, NFkB (complex), PI3K (complex), PPARG, PRDM1, TNF, TP53 | expression |
|  | PPARG | Mapk, PPARG | inhibition |
|  | PPARG | TNF | localization |
|  | PPARG | PPARG | molecular cleavage |
|  | PPARG | Akt, ERK1/2, PPARG | phosphorylation |
|  | PPARG | CPT1B, PPARG | protein-DNA interactions |
|  | PPARG | ADAM22, KLF5, NFkB (complex), PPARG | protein-protein interactions |
|  | PPARG | PPARG | regulation of binding |
|  | PPARG | CPT1B, PPARG | transcription |
|  | PPARG | PPARG | translocation |
|  | PRDM1 | LRRN1, PRDM1, TNF | expression |
|  | PRDM1 | TNF | localization |
|  | PRDM1 | PRDM1 | protein-protein interactions |
|  | PRDM1 | PRDM1 | transcription |
|  | PROM1 | Akt | activation |
|  | PROM1 | ABCB5 | expression |
|  | PROM1 | Akt | phosphorylation |
|  | PROM1 | ERK1/2 | protein-protein interactions |
|  | PROM1 | P110 | translocation |
|  | ROBO1 | Mapk | activation |
|  | ROBO1 | DCC | protein-protein interactions |
|  | RPS13 | DCC | protein-protein interactions |
|  | RTN4R | Akt, ERK1/2 | activation |
|  | RTN4R | TNF | localization |
|  | RTN4R | Akt, ERK1/2 | phosphorylation |
|  | RTN4R | ADAM22, MAG, RTN4R | protein-protein interactions |
|  | RTN4R | MAG, RTN4R | regulation of binding |
|  | SERPINB8 | PLXDC2 | protein-protein interactions |
|  | SERPIND1 | KLF5 | expression |
|  | SERPIND1 | SERPIND1 | protein-protein interactions |
|  | SIAH2 | SIAH2, TP53 | activation |
|  | SIAH2 | DCC, SIAH2 | expression |
|  | SIAH2 | TP53 | modification |
|  | SIAH2 | DCC, SIAH2 | molecular cleavage |
|  | SIAH2 | SIAH2 | phosphorylation |
|  | SIAH2 | DCC, PPARG, SIAH2 | protein-protein interactions |
|  | SIAH2 | SIAH2 | ubiquitination |
|  | SLC24A3 | Ca2+ | chemical-protein interactions |
|  | THY1 | Ca2+ | expression |
|  | THY1 | THY1 | protein-protein interactions |
|  | TLR1-TLR2 | NFkB (complex) | activation |
|  | TLR1-TLR2 | TLR2 | membership |
|  | TLR2 | Akt, ERK1/2, Mapk, NFkB (complex), PI3K (complex) | activation |
|  | TLR2 | NFkB (complex), TLR2, TNF | expression |
|  | TLR2 | Ca2+, NFkB (complex), PI3K (complex), TNF | localization |
|  | TLR2 | Akt, ERK1/2 | phosphorylation |
|  | TLR2 | LGR4, TLR2 | protein-protein interactions |
|  | TLR2 | TLR2 | regulation of binding |
|  | TLR2 | TNF | transcription |
|  | TLR2 | NFkB (complex) | translocation |
|  | TNF | Akt, EIF2A, ERBB2, ERK1/2, Mapk, NFkB (complex), PI3K (complex), PPARG, TLR2, TP53 | activation |
|  | TNF | ADORA2B, Akt, CPT1B, Ca2+, DUSP10, ERBB2, FGF18, KLF5, Mapk, NCAM1, NFkB (complex), NUMB, P110, PDGFA, PLXDC2, PPARG, PRDM1, ROBO1, RPS13, SERPINB8, SERPIND1, THY1, TLR2, TNF, TP53, WNT10A | expression |
|  | TNF | NFkB (complex) | inhibition |
|  | TNF | Ca2+, NFkB (complex), TNF, TP53 | localization |
|  | TNF | DUSP10 | modification |
|  | TNF | ERBB2, ERK1/2, PPARG | molecular cleavage |
|  | TNF | Akt, EIF2A, ERBB2, ERK1/2, Mapk, NFkB (complex), TP53 | phosphorylation |
|  | TNF | NEO1, TNF | protein-protein interactions |
|  | TNF | ARFGAP3, Akt, BABAM2, NFkB (complex), PPARG, TLR2, TNF, TP53 | regulation of binding |
|  | TNF | NFkB (complex), PPARG, TLR2, TNF, | transcription |
|  | TNF | Akt, NFkB (complex), TNF, TP53 | translocation |
|  | TP53 | Akt, ERBB2, ERK1/2, GPR87, Mapk, NFkB (complex), PI3K (complex), TP53 | activation |
|  | TP53 | Ca2+ | chemical-protein interactions |
|  | TP53 | ADORA2B, CGREF1, CPT1B, Ca2+, DCK, FGFBP1, GDA, GPR87, NEO1, PARD6G, PDGFA, PPARG, PRDM1, PROM1, ROBO1, THY1, TNF, TP53, UNC5B, WNT10A, miR-145-5p (and other miRNAs w/seed UCCAGUU), mir-142 | expression |
|  | TP53 | NFkB (complex), PPARG, TP53 | inhibition |
|  | TP53 | Ca2+, NFkB (complex), TNF, TP53 | localization |
|  | TP53 | TP53 | modification |
|  | TP53 | TP53 | molecular cleavage |
|  | TP53 | Akt, ERK1/2, TP53 | phosphorylation |
|  | TP53 | CGREF1, PARD6G, TP53, UNC5B | protein-DNA interactions |
|  | TP53 | BABAM2, BRINP1, ENAH, ERBB2, ERK1/2, GMPS, KLF5, NUMB, RPS13, TP53, | protein-protein interactions |
|  | TP53 | Ca2+, KLF5, NFkB (complex), TP53 | regulation of binding |
|  | TP53 | ERBB2, GPR87, PODXL, TP53, miR-145-5p (and other miRNAs w/seed UCCAGUU) | transcription |
|  | TP53 | TP53 | translocation |
|  | TP53 | NUMB, TP53 | ubiquitination |
|  | TRIM40 | NFkB (complex) | activation |
|  | TRIM40 | LGR4 | protein-protein interactions |
|  | TRMO | DCC ERBB2 | protein-protein interactions |
|  | UNC5B | TNF | expression |
|  | UNC5B | DCC, NEO1, NTN4, UNC5B | protein-protein interactions |
|  | miR-145-5p (and other miRNAs w/seed UCCAGUU) | KLF5, NTN4, PODXL | RNA-RNA interactions: microRNA targeting |
|  | miR-145-5p (and other miRNAs w/seed UCCAGUU) | KLF5, NTN4, PODXL | expression |
|  | miR-145-5p (and other miRNAs w/seed UCCAGUU) | TNF | localization |
|  | mir-142 | PROM1, TNF | expression |
| Figure 5 |  |  |  |
|  | ADORA3 | ERK1/2, NFkB (complex) | activation |
|  | ADORA3 | SPP1 | expression |
|  | ADORA3 | IFNG, IL12 (complex) | localization |
|  | ADORA3 | ERK1/2 | phosphorylation |
|  | ALKBH5 | ADGRG2, CAV1, LAMA1 | expression |
|  | AP4B1 | NFkB (complex) | activation |
|  | AP4B1 | AP4S1 | protein-protein interactions |
|  | AP4S1 | AP4B1 | protein-protein interactions |
|  | APP | APP, ERK1/2, NFkB (complex), PI3K (complex) | activation |
|  | APP | ADORA3, APP, CTNNB1, ERBB2, IFNG, NFkB (complex), POU4F1, TGFB1, TOP2A | expression |
|  | APP | APP, CTNNB1, IL12 (complex), NFkB (complex) | localization |
|  | APP | APP | modification |
|  | APP | APP | molecular cleavage |
|  | APP | APP, ERK1/2 | phosphorylation |
|  | APP | ADAM22, AP4S1, APP, CTNNB1, TGFB1 | protein-protein interactions |
|  | APP | APP, NFkB (complex) | regulation of binding |
|  | APP | APP | translocation |
|  | ARHGAP28 | NFkB (complex) | activation |
|  | CAV1 | CAV1, ERK1/2, Mek, NFkB (complex), PI3K (complex) | activation |
|  | CAV1 | ADGRG2, CAV1, CTNNB1, ERK1/2, IFNG | expression |
|  | CAV1 | CTNNB1 | localization |
|  | CAV1 | CAV1, ERK1/2, Mek, PI3K (complex) | phosphorylation |
|  | CAV1 | APP | protein-RNA interactions |
|  | CAV1 | CAV1, CTNNB1, PI3K (complex) | protein-protein interactions |
|  | CAV1 | CAV1, CTNNB1 | regulation of binding |
|  | CAV1 | CAV1 | transcription |
|  | CAV1 | CAV1 | translocation |
|  | CDKL2 | APP | protein-protein interactions |
|  | CMTM3 | APP | protein-protein interactions |
|  | CTNNB1 | CTNNB1, Mek, NFkB (complex), TCF | activation |
|  | CTNNB1 | ADGRG2, APP, CTNNB1, IFNG. ISL1, SPP1, TGFB1 | expression |
|  | CTNNB1 | CTNNB1, IFNG, IL12 (complex) | localization |
|  | CTNNB1 | CTNNB1 | molecular cleavage |
|  | CTNNB1 | CTNNB1, Mek | phosphorylation |
|  | CTNNB1 | SPP1 | protein-DNA interactions |
|  | CTNNB1 | APP, CAV1, CTNNB1, NFkB (complex), TCF | protein-protein interactions |
|  | CTNNB1 | CTNNB1, TCF | regulation of binding |
|  | CTNNB1 | SPP1, TCF | transcription |
|  | CTNNB1 | CTNNB1 | translocation |
|  | CTNNB1 | CTNNB1 | ubiquitination |
|  | DCTN6 | APP | protein-protein interactions |
|  | ERBB2 | CAV1, CTNNB1, ERBB2, ERK1/2, NFkB (complex), PI3K (complex), TCF | activation |
|  | ERBB2 | ADORA3, CTNNB1, ERBB2, GSTZ1, HEPH, IFNG, NFkB (complex), SPARCL1, TOP2A | expression |
|  | ERBB2 | LAMA3, PODXL | localization |
|  | ERBB2 | ERBB2 | molecular cleavage |
|  | ERBB2 | CAV1, CTNNB1, ERBB2, ERK1/2 | phosphorylation |
|  | ERBB2 | CTNNB1, ERBB2, PI3K (complex) | protein-protein interactions |
|  | ERBB2 | ERBB2, NFkB (complex) | regulation of binding |
|  | ERBB2 | TOP2A | transcription |
|  | ERBB2 | ERBB2 | ubiquitination |
|  | ERK1/2 | CTNNB1, ERK1/2, NFkB (complex), TCF | activation |
|  | ERK1/2 | IFNG, IL12 (complex), SPP1 | expression |
|  | ERK1/2 | APP, TGFB1 | localization |
|  | ERK1/2 | ERK1/2, NFkB (complex) | phosphorylation |
|  | ERK1/2 | ERK1/2 | protein-protein interactions |
|  | ERK1/2 | ERK1/2 | regulation of binding |
|  | ERK1/2 | CAV1 | transcription |
|  | ERK1/2 | ERK1/2 | translocation |
|  | FRY | CDKL2 | protein-protein interactions |
|  | GSTZ1 | GSTZ1 | protein-protein interactions |
|  | GSTZ1 | GSTZ1 | regulation of binding |
|  | Gpcr | ERK1/2, NFkB (complex), PI3K (complex) | activation |
|  | Gpcr | Gpcr | inhibition |
|  | Gpcr | ADGRG2, ADORA3 | membership |
|  | Gpcr | ERK1/2, Gpcr | phosphorylation |
|  | Gpcr | Gpcr | regulation of binding |
|  | HEPH | HEPH | expression |
|  | HNRNPA2B1 | ADGRG2, HNRNPA2B1, IFNG | expression |
|  | HNRNPA2B1 | IFNG | localization |
|  | HNRNPA2B1 | CTNNB1, HNRNPA2B1 | protein-protein interactions |
|  | IFNG | ERK1/2, IFNG, Mek, NFkB (complex), PI3K (complex) | activation |
|  | IFNG | chondroitin sulfate B | chemical-protein interactions |
|  | IFNG | ADGRG2, APP, CAV1, CTNNB1, ERBB2, IFNG, IL12 (complex), ISL1, NFkB (complex), SPP1, TGFB1 | expression |
|  | IFNG | NFkB (complex) | inhibition |
|  | IFNG | APP, IFNG, IL12 (complex), NFkB (complex) | localization |
|  | IFNG | APP | molecular cleavage |
|  | IFNG | ERK1/2 | phosphorylation |
|  | IFNG | IFNG | protein-protein interactions |
|  | IFNG | CTNNB1, ERK1/2, NFkB (complex) | regulation of binding |
|  | IFNG | IFNG | transcription |
|  | IFNG | ERK1/2, IFNG, NFkB (complex) | translocation |
|  | IL12 (complex) | ERK1/2, IFNG, NFkB (complex) | activation |
|  | IL12 (complex) | GCNT2, IFNG, IL12 (complex), NFkB (complex), TGFB1 | expression |
|  | IL12 (complex) | IFNG, IL12 (complex) | localization |
|  | IL12 (complex) | IFNG | molecular cleavage |
|  | IL12 (complex) | IFNG | regulation of binding |
|  | IL12 (complex) | IFNG | transcription |
|  | IL12 (complex) | IL12 (complex) | translocation |
|  | ISL1 | ADGRG2, IFNG, IL12 (complex), POU4F1 | expression |
|  | ISL1 | APP, ISL1 | protein-protein interactions |
|  | Irp | APP, HEPH | expression |
|  | Irp | APP | protein-RNA interactions |
|  | KMT2D | ADGRG2 | expression |
|  | KMT2D | CTNNB1, KMT2D | protein-protein interactions |
|  | KMT2D | KMT2D | regulation of binding |
|  | LAMA1 | ERK1/2 | activation |
|  | LAMA1 | ERK1/2 | phosphorylation |
|  | LAMA1 | AP4B1, AP4S1, DCTN6, LAMA3 | protein-protein interactions |
|  | LAMA3 | ERK1/2 | activation |
|  | LINC00461 | ERK1/2 | activation |
|  | LINC00461 | TOP2A | expression |
|  | LINC00461 | ERK1/2 | phosphorylation |
|  | LRRN1 | ERK1/2 | activation |
|  | Mek | CTNNB1, ERK1/2, Mek, NFkB (complex) | activation |
|  | Mek | CTNNB1, ERBB2, IFNG, IL12 (complex), SEMA6A | expression |
|  | Mek | APP, ERK1/2 | localization |
|  | Mek | ERK1/2, Mek | phosphorylation |
|  | Mek | ERBB2, ERK1/2 | protein-protein interactions |
|  | Mek | TOP2A | transcription |
|  | NFkB (complex) | NFkB (complex), PI3K (complex), | activation |
|  | NFkB (complex) | APP, CTNNB1, ERK1/2, IFNG, IL12 (complex), ISL1, SPP1, TGFB1 | expression |
|  | NFkB (complex) | APP, CTNNB1, IL12 (complex), TGFB1 | localization |
|  | NFkB (complex) | ERBB2 | molecular cleavage |
|  | NFkB (complex) | ERBB2, IFNG, SPP1 | protein-DNA interactions |
|  | NFkB (complex) | CTNNB1 | protein-protein interactions |
|  | NFkB (complex) | NFkB (complex) | regulation of binding |
|  | NFkB (complex) | IFNG | transcription |
|  | NFkB (complex) | SPP1 | transcription |
|  | NFkB (complex) | NFkB (complex) | translocation |
|  | OGA | ADGRG2, CAV1, CTNNB1, TGFB1 | expression |
|  | OGA | CTNNB1 | phosphorylation |
|  | OGA | CTNNB1 | regulation of binding |
|  | PI3K (complex) | CTNNB1, ERK1/2, Mek, NFkB (complex), PI3K (complex) | activation |
|  | PI3K (complex) | FRY, IFNG, IL12 (complex), NFkB (complex), SPP1 | expression |
|  | PI3K (complex) | APP, CTNNB1, IL12 (complex) | localization |
|  | PI3K (complex) | CTNNB1 | molecular cleavage |
|  | PI3K (complex) | CTNNB1, ERK1/2, NFkB (complex), PI3K (complex) | phosphorylation |
|  | PI3K (complex) | CAV1, CTNNB1, ERBB2 | protein-protein interactions |
|  | PI3K (complex) | CTNNB1, NFkB (complex), PI3K (complex) | regulation of binding |
|  | PI3K (complex) | IFNG, NFkB (complex) | transcription |
|  | PODXL | PI3K (complex) | activation |
|  | POU4F1 | ADGRG2, POU4F1 | expression |
|  | RIMS4 | APP | protein-protein interactions |
|  | SEMA6A | ERK1/2 | activation |
|  | SEMA6A | ERK1/2 | phosphorylation |
|  | SLC39A6 | ERK1/2 | activation |
|  | SPP1 | CTNNB1, ERK1/2, Mek, NFkB (complex), PI3K (complex) | activation |
|  | SPP1 | ERBB2, ERK1/2, GSTZ1, IFNG, IL12 (complex), SEMA6C, SPARCL1, SPP1, TGFB1 | expression |
|  | SPP1 | IFNG, SPP1, TGFB1 | localization |
|  | SPP1 | SPP1 | molecular cleavage |
|  | SPP1 | ERK1/2 | phosphorylation |
|  | SPP1 | CMTM3, RIMS4, SLC39A6 | protein-protein interactions |
|  | SPP1 | NFkB (complex), SPP1 | regulation of binding |
|  | TCF | CTNNB1 | activation |
|  | TCF | ADGRG2, CTNNB1, TCF | protein-protein interactions |
|  | TCF | TCF | regulation of binding |
|  | TEPSIN | AP4B1, AP4S1, APP, LAMA1, TEPSIN | protein-protein interactions |
|  | TGFB1 | CTNNB1, ERBB2, ERK1/2, Mek, NFkB (complex), PI3K (complex), TGFB1 | activation |
|  | TGFB1 | ADGRG2, APP, CAV1, CTNNB1, ERK1/2, IFNG, IL12 (complex), OGA, PODXL, POU4F1, SPARCL1, SPP1, TGFB1, chondroitin sulfate B, pyrophosphate | expression |
|  | TGFB1 | NFkB (complex), TOP2A | inhibition |
|  | TGFB1 | APP, CTNNB1, ERK1/2, IFNG, IL12 (complex), TGFB1, beta-estradiol, pyrophosphate | localization |
|  | TGFB1 | chondroitin sulfate B | modification |
|  | TGFB1 | APP, CTNNB1, NFkB (complex) | molecular cleavage |
|  | TGFB1 | CTNNB1, ERK1/2, Mek, PI3K (complex) | phosphorylation |
|  | TGFB1 | APP, ERBB2, TGFB1 | protein-protein interactions |
|  | TGFB1 | CTNNB1, ERBB2, NFkB (complex), TGFB1 | regulation of binding |
|  | TGFB1 | CTNNB1, SPP1, TGFB1, TOP2A, | transcription |
|  | TGFB1 | CTNNB1 | translocation |
|  | TOP2A | TOP2A | expression |
|  | TOP2A | CTNNB1, TOP2A | protein-protein interactions |
|  | beta-estradiol | APP, CTNNB1, ERBB2, ERK1/2, Gpcr, NFkB (complex), PI3K (complex) | activation |
|  | beta-estradiol | ERBB2 | chemical-protein interactions |
|  | beta-estradiol | ADGRG2, APP, CAV1, CDKL2, CTNNB1, ERBB2, IFNG, SLC39A6, TGFB1, TOP2A | expression |
|  | beta-estradiol | NFkB (complex), PI3K (complex), | inhibition |
|  | beta-estradiol | APP, IFNG, KMT2D, TGFB1, beta-estradiol | localization |
|  | beta-estradiol | APP | molecular cleavage |
|  | beta-estradiol | CTNNB1, ERK1/2 | phosphorylation |
|  | beta-estradiol | CAV1, ERBB2, KMT2D, NFkB (complex), PI3K (complex), beta-estradiol | regulation of binding |
|  | beta-estradiol | CAV1, ERBB2, | transcription |
|  | beta-estradiol | beta-estradiol | translocation |
|  | chondroitin sulfate B | ERK1/2, NFkB (complex) | activation |
|  | chondroitin sulfate B | APP | chemical-protein interactions |
|  | chondroitin sulfate B | ERK1/2 | phosphorylation |
|  | chondroitin sulfate B | LAMA1 | regulation of binding |
|  | pyrophosphate | PI3K (complex) | activation |
|  | pyrophosphate | SPP1 | expression |
